# A Meta-Analysis of Quantitative Trait Loci Associated with Multiple Disease Resistance in Rice (*Oryza sativa* L.)

**DOI:** 10.3390/plants9111491

**Published:** 2020-11-05

**Authors:** Ilakiya Sharanee Kumar, Kalaivani Nadarajah

**Affiliations:** Department of Biological Sciences and Biotechnology, Faculty of Science and Technology, Universiti Kebangsaan Malaysia, Bangi 43000, Malaysia

**Keywords:** rice (*Oryza sativa* L.), rice blast (*Magnaporthe oryzae*), sheath blight (*Rhizoctonia solani*), bacterial leaf blight (*Xanthomonas oryzae*), QTL, consensus map, meta-QTL, broad spectrum resistance, defense genes, R-genes

## Abstract

Rice blast, sheath blight and bacterial leaf blight are major rice diseases found worldwide. The development of resistant cultivars is generally perceived as the most effective way to combat these diseases. Plant disease resistance is a polygenic trait where a combinatorial effect of major and minor genes affects this trait. To locate the source of this trait, various quantitative trait loci (QTL) mapping studies have been performed in the past two decades. However, investigating the congruency between the reported QTL is a daunting task due to the heterogeneity amongst the QTLs studied. Hence, the aim of our study is to integrate the reported QTLs for resistance against rice blast, sheath blight and bacterial leaf blight and objectively analyze and consolidate the location of QTL clusters in the chromosomes, reducing the QTL intervals and thus identifying candidate genes within the selected meta-QTL. A total of twenty-seven studies for resistance QTLs to rice blast (8), sheath blight (15) and bacterial leaf blight (4) was compiled for QTL projection and analyses. Cumulatively, 333 QTLs associated with rice blast (114), sheath blight (151) and bacterial leaf blight (68) resistance were compiled, where 303 QTLs could be projected onto a consensus map saturated with 7633 loci. Meta-QTL analysis on 294 QTLs yielded 48 meta-QTLs, where QTLs with membership probability lower than 60% were excluded, reducing the number of QTLs within the meta-QTL to 274. Further, three meta-QTL regions (MQTL2.5, MQTL8.1 and MQTL9.1) were selected for functional analysis on the basis that MQTL2.5 harbors the highest number of QTLs; meanwhile, MQTL8.1 and MQTL9.1 have QTLs associated with all three diseases mentioned above. The functional analysis allows for determination of enriched gene ontology and resistance gene analogs (RGAs) and other defense-related genes. To summarize, MQTL2.5, MQTL8.1 and MQTL9.1 have a considerable number of R-genes that account for 10.21%, 4.08% and 6.42% of the total genes found in these meta-QTLs, respectively. Defense genes constitute around 3.70%, 8.16% and 6.42% of the total number of genes in MQTL2.5, MQTL8.1 and MQTL9.1, respectively. This frequency is higher than the total frequency of defense genes in the rice genome, which is 0.0096% (167 defense genes/17,272 total genes). The integration of the QTLs facilitates the identification of QTL hotspots for rice blast, sheath blight and bacterial blight resistance with reduced intervals, which helps to reduce linkage drag in breeding. The candidate genes within the promising regions could be utilized for improvement through genetical engineering.

## 1. Introduction

Rice blast (RB), sheath blight (SHB) and bacterial leaf blight (BLB) are the major rice diseases reported in rice. Rice blast is the number one destructive disease, followed by sheath blight and bacterial leaf blight. The former two diseases are generally caused by fungal pathogens, *Magnaporthe oryzae* and *Rhizoctonia solani*, respectively, while the latter is caused by a bacterial pathogen, *Xanthomonas oryzae.* Rice blast can cause up to 30% reduction in yield annually and, under favorable conditions, the losses can be up to 100% [1]. Meanwhile, for sheath blight, the reduction in yield can be up to 42% [2], and for bacterial leaf blight, the losses can be up to 60% [3]. Continuous effort has been undertaken to keep these diseases under control by encouraging farmers to exercise good farming practices. While fungicides are the main method of controlling disease, their detrimental effects on the environment and the handlers cannot be discounted. This has caused the rice industry to be largely dependent on the generation of new resistant varieties as it is perceived to be the most efficient way by far.

Exploitation of the genetic determinants will improve other susceptible varieties. Taking this into account, breeders and biotechnologists are striving to locate the source of resistance to understand and utilize the genetics underlying this process. The emergence of molecular markers such as simple sequence repeats (SSR) and single nucleotide polymorphism (SNP) are amenable for large-scale screening in breeding. It also provides a useful avenue to map genes or quantitative trait loci (QTL) responsible for a particular trait.

There has been a large number of QTL mapping studies for disease resistance against rice blast, sheath blight and bacterial leaf blight [4,5,6,7,8,9,10,11,12,13,14,15,16,17,18,19,20,21,22,23,24,25,26,27,28,29,30,31]. However, these studies are conditioned to various breeding methods and different QTL mapping analyses, which makes it difficult to handpick suitable QTL candidates for breeding programs that carry multiple resistance. In view of this, it would be interesting if these QTLs could be presented in an integrated manner with an exhaustive analysis to evaluate the reported QTLs. To accomplish this, a structured, statistical method that takes into account the parameters used in previous experiments is required to provide robust, viable and stable target QTLs. In an effort to breed for multiple disease resistance, pyramiding of multiple QTLs for different diseases has been conducted by breeders [32,33]. However, this method can be quite a lengthy process as it begins with combining two QTLs for two different diseases into one line and then, as the generation is established, another QTL is added to achieve the desirable traits. Moreover, the QTLs that are introgressed may have epistatic interaction, which may affect the outcome of the introgression process. However, if there are incidences where QTLs for different diseases coincide, the attempt to introgress multiple QTLs can be avoided. To observe such incidences, all the QTLs reported for the diseases should be presented in an integrated manner and mapped out to see if there is any co-localization of QTLs.

Meta-QTL analysis has the ability to integrate information from various QTL mapping studies, enabling a high degree of statistical power over the exorbitant amount of data. This particular method is made possible through several software programs, such as Meta-QTL and BioMercator V4.2, where specific sets of algorithms are formulated and embedded for precise evaluation and recalculation of the genetic position for any given QTL [34]. The generation of a consensus map and the subsequent projection of QTL allows for the identification of regions that are heavily populated with the QTL for any given trait. On top of this, new genetic positions that agree with all the maps from the previous experiments are also derived. Although each software program includes the same set of algorithms, BioMercator V4.2 is the most advanced as compared to the former program, which is not user-friendly and requires command lines to conduct each analytical function in meta-QTL analysis. Further, almost all the previous meta-QTL studies used BioMercator V4.2. This would make comparison between studies easier.

The extensive use of meta-QTL analysis demonstrates the importance of this method. Said et al. (2019) accumulated 1223 QTLs associated with fiber quality, yield, yield-related and morphological traits, drought tolerance and disease resistance from 42 different QTL studies in cotton (*Gossypium* spp.) and identified putative QTL clusters through meta-QTL analysis [35]. Similarly, Swamy et al. (2011) conducted a meta-QTL analysis on 15 studies involving QTL associated with grain yield during drought in rice and identified 14 meta-QTLs with reduced genetic intervals from 53 individual QTLs [36]. A meta-analysis on 12 QTLs studied for salt tolerance at seedling stage was conducted, where 11 meta-QTLs with reduced genetic intervals were mapped [37]. However, meta-QTL analysis for disease resistance QTL in rice has not been reported. These meta-QTLs will be useful for the breeding of multiple disease resistance in rice. The markers that closely flank the meta-QTL region can be used for the screening of resistant progenies through marker-assisted selection to circumvent the usual conventional screening process.

## 2. Results and Discussion

### 2.1. Compilation and Characterization of QTL Studies Involving Sheath Blight, Rice Blast and Bacterial Leaf Blight Resistance

An exhaustive search against the published papers on QTL mapping enabled us to compile 27 different studies (15 SHB, 8 RB, 4 BLB) for the generation of a consensus map and meta-QTL analysis (Table 1). These 27 studies varied in the form of markers, parents, population size and method of breeding. Various types of markers, such as simple sequence repeat (SSR), restriction fragment length polymorphism (RFLP), and insertion and deletions (InDels), were used to build the linkage maps in these studies. The number of markers used ranged from 62 to 279, at an average of 141 markers per study. In addition, the population size used for screening was between 82 and 1200 progenies. Some of these studies had to be excluded as they lacked important information such as the genetic positions of the QTL and markers, phenotypic variance and logarithm of odds (LOD). Some excluded studies did not have any common markers with the rest of the studies compiled. After careful examination of the QTLs, a total number of 333 QTLs associated with sheath blight, rice blast and bacterial leaf blight resistance were compiled and prepared as individual input files for each study and subjected to mapping, QTL projection and meta-QTL analysis. The compilation of 333 QTLs does not necessarily cover all the QTL published. The results obtained are the best representation of what could be obtained to our maximum potential.

### 2.2. Generation of Consensus Map and Projection of QTLs

**Figure 1:**
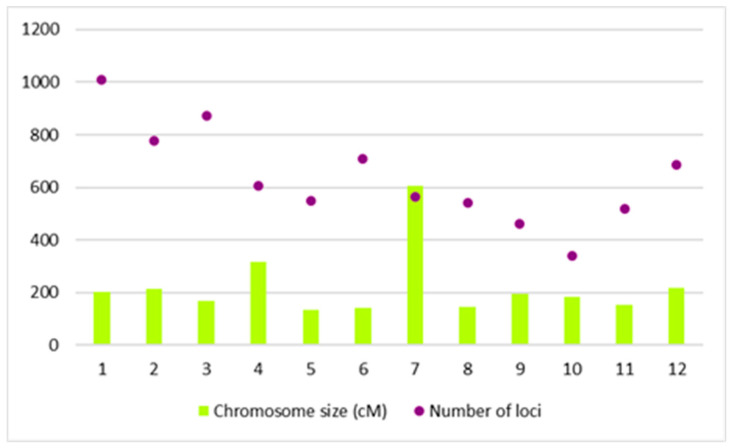
Bar chart showing the genetic size of the consensus map along with the number of loci mapped in the 12 chromosomes. Chromosome 7 has the largest map size, while chromosome 1 has the highest number of loci mapped.

The iterative projection of the maps along with the QTL onto the reference map [39] resulted in a total number of 7633 loci composed of markers and some important genes. Figure 1 presents the genetic map size and the number of loci mapped in each chromosome. The map size of the chromosomes ranged from 134.4 to 604.77cM. The iterative map carefully removed any studies or QTLs that had inverted or unlikely positions as compared to the reference map to avoid any discrepancy in generating the consensus map. This resulted in a reduction of the total number of QTLs mapped to 303 QTLs (from 333 QTLs). The highest number of QTLs is attributed to chromosome 3 with 42 QTLs, followed closely by chromosome 2 and 1 with 40 and 39 QTLs, respectively. Chromosome 10 has the lowest number of QTLs (eight QTLs), which is expected to be owing to the smallest size of this chromosome (22.4 MB) [40].

### 2.3. Meta-QTL for Sheath Blight, Rice Blast and Bacterial Leaf Blight Resistance

A great number of QTL studies have been conducted over the past few years to help pinpoint regions with significant associations with a desired trait. In addition to the overwhelming amount of data, which is tedious to browse through, the discrepancies that exist between these studies make the analyses even more complicated. Since the introduction of meta-analysis for QTLs back in 2000 [41], a spurt of meta-analysis studies for QTL in various organisms were observed in subsequent years. The earliest reports of QTL meta-analysis in rice were documented for blast resistance, drought tolerance and root architecture, while rice grain yield under drought was studied years later. Most recently, meta-analyses on QTL associated with seedling-stage salt tolerance and panicle-related traits in rice were also performed.

In this study, we have conducted meta-QTL analysis on QTLs associated with resistance against rice blast, sheath blight and bacterial leaf blight to investigate the consensus between these diseases and to refine the QTL clusters. The previously published meta-analysis on QTL and genes in rice blast resistance was performed on a physical map and the meta-QTLs were obtained through the method devised by Gerber and Goffinet [42]. In contrast, the present study made use of a more advanced method by Veyrieras, which is performed on a genetic map. This method allows for the detection of more than four clusters, in contrast to the Gerber and Goffinet method, which limits the meta-QTLs to four clusters.

**Figure 2:**
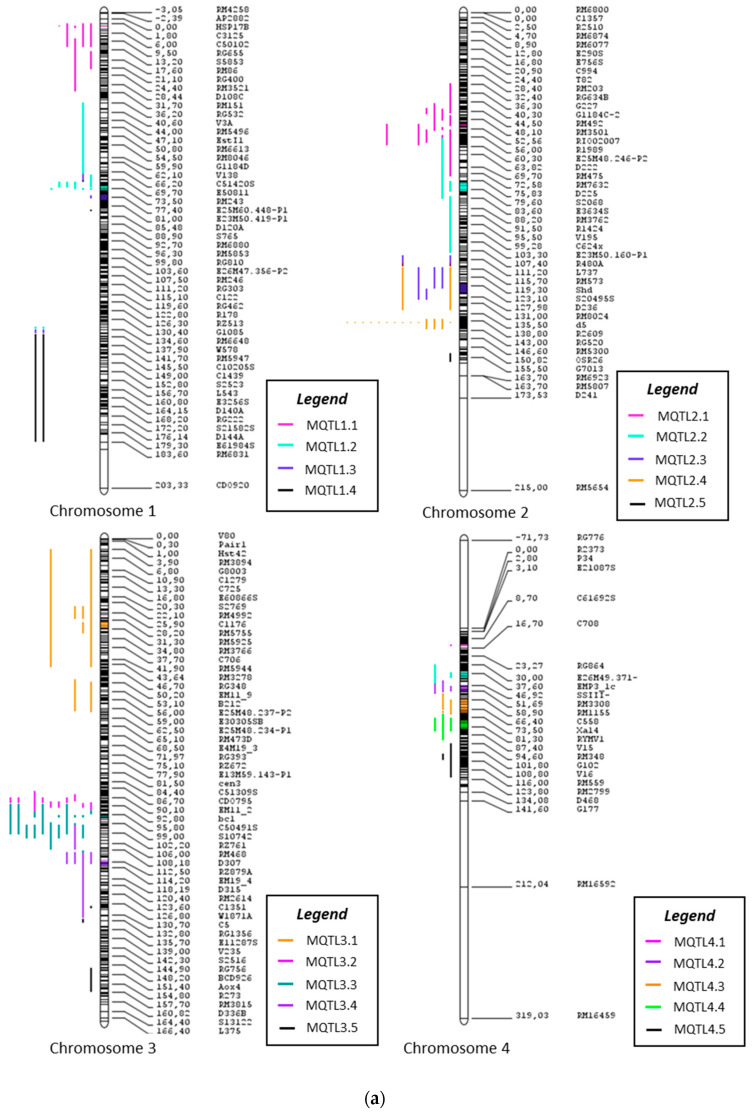

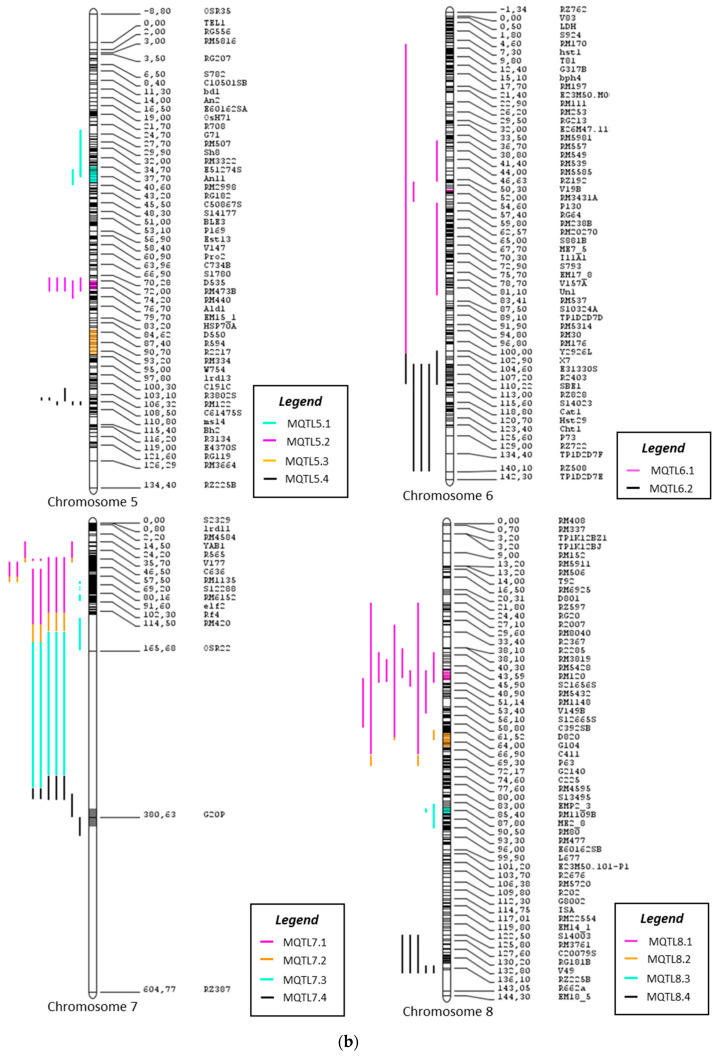

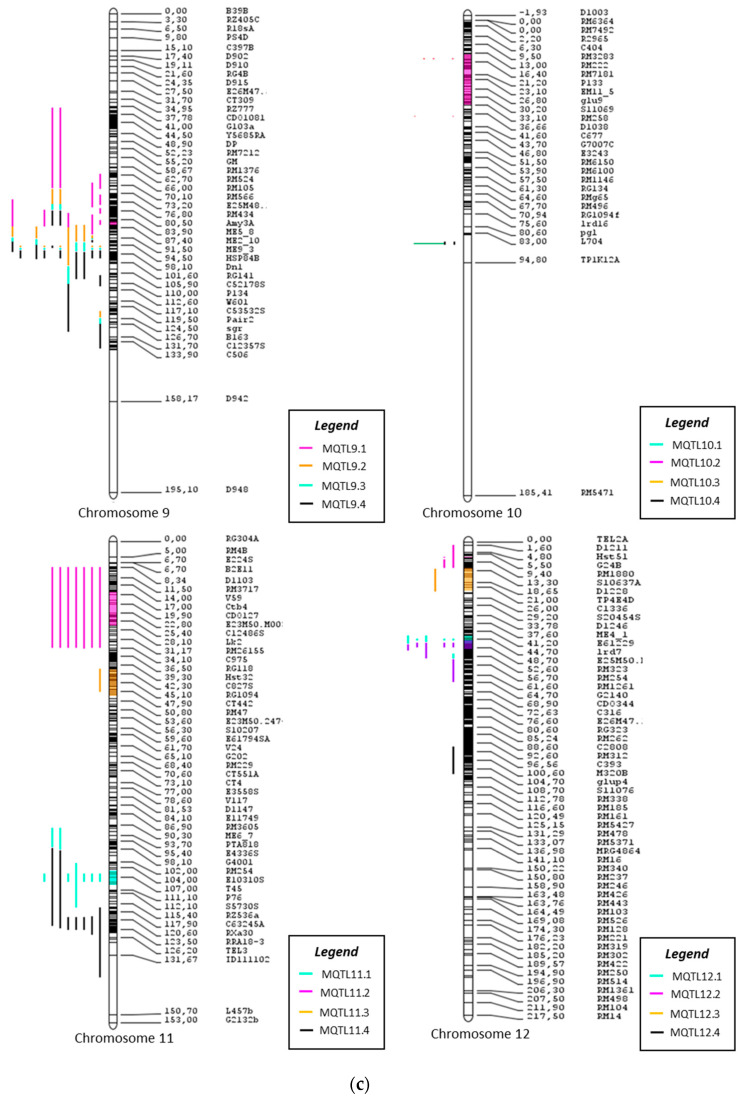
The consensus map of the QTLs associated with RB, SHB and BLB resistance following meta-analysis, (**a**) chromosomes 1–4; (**b**) chromosomes 5–8; (**c**) chromosomes 9–12. The colored regions represent the meta-QTL regions with reduced confidence intervals. The QTL are colored according to their respective meta-QTL regions.

From 303 QTLs projected in the current study, only 294 QTLs could be assigned to their respective meta-QTL clusters following meta-QTL analysis. The consensus map that was subjected to meta-analysis is represented in Figure 2. A total of 48 meta-QTLs were discovered, with confidence intervals ranging from 0 to 23.94cM. Meanwhile, the LOD and r2 of the initial QTL vary from 0.87 to 36.7% and 0.2% to 67.9% respectively. These meta-QTL regions were screened further based on the following criteria: [40] (1) the membership probability of the QTL assigned to the respective meta-QTL should be >60%, (2) the meta-QTL region should at least include two QTLs with the aforementioned membership probabilities, and (3) the lowest number of QTLs must come from independent studies [43].

In chromosome 1, 36 QTLs provided the basis for four meta-QTLs. Twelve (12) QTLs were excluded as their membership probability was <60%. MQTL1.1 had the highest number of QTLs (11) associated with sheath blight and rice blast in chromosome 1. Chromosome 2, with the highest number of QTLs projected (40 QTLs), had a total of six meta-QTLs. Nevertheless, two meta-QTLs had to be discarded as they did not meet the criteria. MQTL2.5 had the highest number of QTLs associated with sheath blight and rice blast resistance (19 QTLs) as compared to all meta-QTLs in all chromosomes. In total, five meta-QTLs were identified in chromosome 3 from the initial 35 QTLs after excluding QTLs with >60% membership probability. MQTL3.3 had the highest number of QTLs (11) linked to resistance against sheath blight and bacterial leaf blight. A total of 14 QTLs were projected in chromosome 4, resulting in the identification of six meta-QTLs. However, three meta-QTLs were excluded as they did not comply with the criteria established. 

Chromosome 5 had a total of 16 QTLs which were assigned to four meta-QTLs. Chromosome 6 had 19 QTLs, which was reduced to 16 QTLs. Around two meta-QTLs were identified, with the highest number of QTLs attributed to MQTL6.1 (12 QTLs), solely contributing to resistance against rice blast. Around four meta-QTLs were identified in chromosome 7, with 17 QTLs reduced from 23 QTLs. MQTL7.1 had 12 QTLs associated with resistance against rice blast, sheath blight and bacterial leaf blight. In chromosome 8, 20 QTLs were clustered into four meta-QTLs. MQTL8.1 had the highest number of QTLs (12 QTLs) associated with rice blast, sheath blight and bacterial leaf blight.

In chromosome 9, four meta-QTLs were identified with 33 QTL projections, which was then reduced to 24. The highest number of QTLs in chromosome 9 was associated with MQTL9.1, with 10 QTLs associated with rice, blast, sheath blight and bacterial leaf blight. We also managed to discover two meta-QTLs in chromosome 10, which had the lowest number of QTLs (six QTLs). Meta-analysis on chromosome 11, with an initial projection of 30 QTLs, which was reduced to 24 QTLs, resulted in four meta-QTLs. The highest number of QTLs belonged to MQTL11.3, with eight QTLs associated with rice blast and bacterial leaf blight. Lastly, five meta-QTLs were discovered in chromosome 12, with 15 QTLs at initial projection and 14 QTLs after exclusion of QTLs with low membership probability.

**Figure 3:**
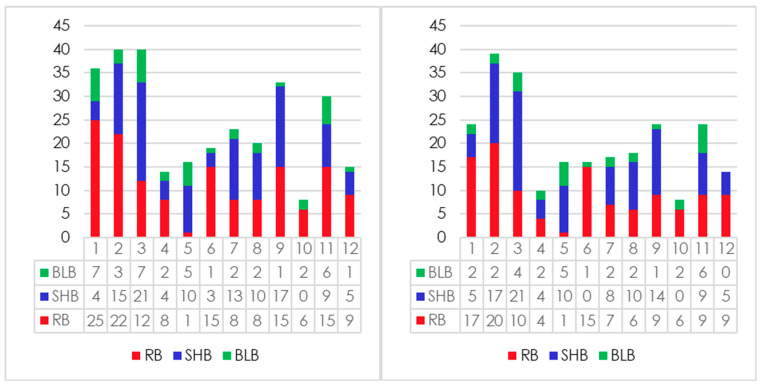
Distribution of QTLs associated with resistance against sheath blight, rice blast and bacterial leaf blight across the chromosomes. The bar chart on the left depicts the number of QTLs assigned after meta-analysis, while the bar chart on the right depicts the number of QTLs after exclusion of QTLs with membership probability <60%.

**Figure 4:**
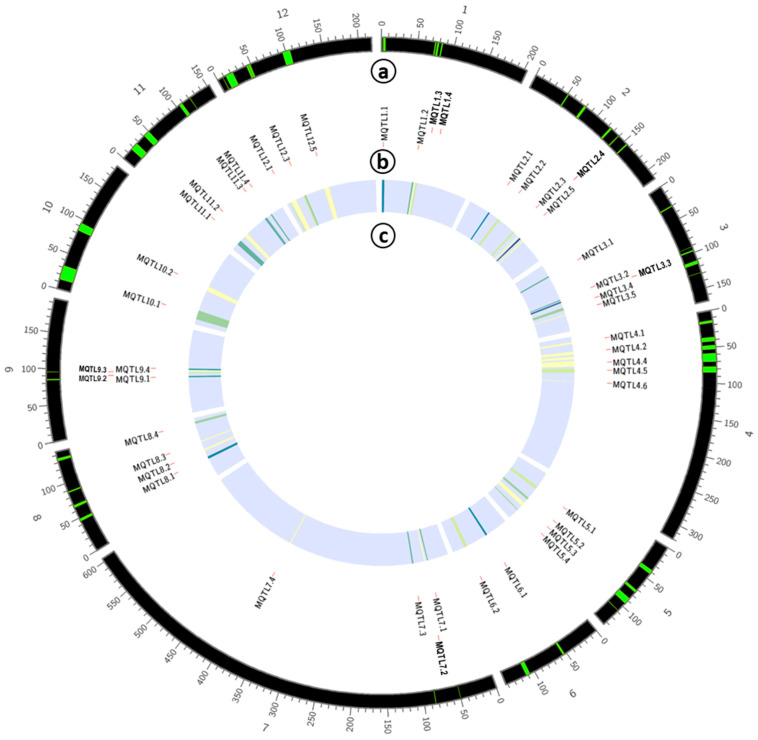
Circular plot representing the overall meta-QTL information where “a” indicates the position of the meta-QTLs in the chromosome, “b” the meta-QTL names and “c” the frequency of QTLs involved for each meta-QTL mapped (the frequency increases from lighter to darker color—yellow to blue).

Based on the observations above, it can be concluded that variation exists in terms of the number of QTLs assigned as members for each meta-QTL. The maximum number of QTLs clustered in one meta was from MQTL2.5, with 19 QTLs associated with RB and SHB resistance. On the other hand, it was also noted that singular QTLs were assigned as one meta-QTL. This was observed with 10 meta-QTLs: MQTL2.4, MQTL2.6, MQTL4.1, MQTL4.3, MQTL4.4, MQTL7.2, MQTL8.2, MQTL9.3, MQTL12.2 and MQTL12.5. Since these regions only had singular QTLs, they were excluded from further elucidation. Further, regions with QTLs from the same study were also excluded (MQTL5.3 and MQTL 10.2), bringing down the total meta-QTLs to 36 (Figure 2 and Figure 3 and Table 2). Figure 4 depicts and summarizes the size and position of meta-QTLs in each chromosome along with the frequency of QTLs assigned for each meta-QTL.

For individual resistance against rice blast, MQTL6.1 and MQTL7.3 seem like the best regions as they accommodated 12 and 7 QTLs for rice blast, respectively. On the other hand, MQTL5.5, MQTL8.4 and MQTL11.1 are the best regions for resistance against sheath blight, with six, five and seven QTLs for sheath blight, respectively. Finally, MQTL5.2 possesses the highest number of QTLs (five QTLs) for resistance against bacterial leaf blight.

Over the years, a substantial amount of resistance genes which are highly specific to certain diseases were discovered. However, these resistance genes are often defeated by the ever-evolving pathogens. In contrast, QTL regions which confer non-specific resistance and are highly durable could provide a “killing two birds with one stone” situation, where it may provide multiple resistance to diseases. For multiple disease resistance, three meta-QTLs, MQTL2.5, MQTL8.1 and MQTL9.1, appear to be excellent regions to breed for broad-spectrum resistance. MQTL2.5 has the highest number of QTLs associated with sheath blight and rice blast, while MQTL8.1 and MQTL9.1 have the highest number of QTLs associated with all three diseases. Given these reasons, these three meta-QTLs were selected as candidates for functional analysis to mine for candidate genes.

### 2.4. Identification of Candidate Genes within the Best Candidate meta-QTL

To discover candidate genes, a functional analysis was performed on best meta-QTL regions. Out of the 41 candidate meta-QTLs identified, we selected the best three meta-QTLs, MQTL2.5, MQTL8.1 and MQTL9.1, for functional analysis. MQTL2.5 was chosen because it has the highest number of QTLs (19 QTLs) compared to other meta-QTLs, which exhibits good potential for breeding against sheath blight and rice blast. Meanwhile, MQTL8.1 and MQTL9.1 have the highest numbers of QTLs associated with resistance to all three diseases, rice blast, sheath blight and bacterial leaf blight, with 12 and 10 QTLs, respectively (Table 2). These two meta-QTLs are the most promising regions as they house the highest number of QTLs associated with multiple disease resistance.

#### 2.4.1. MQTL2.5

**Figure 5:**
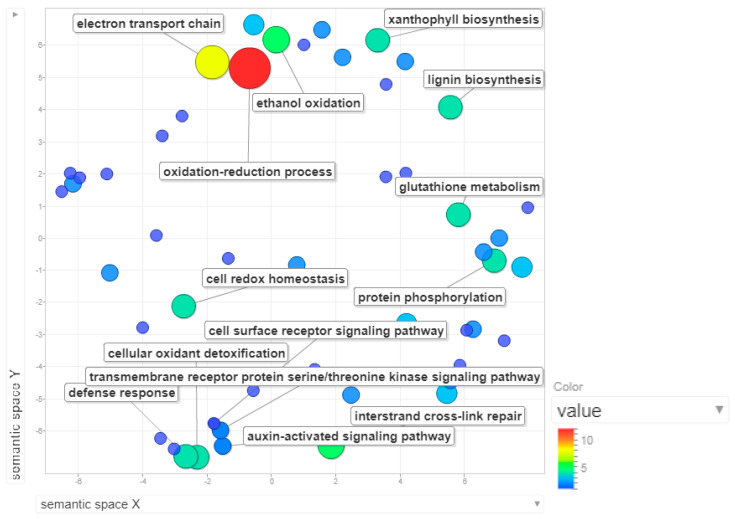
Scatterplot representing the enriched biological process gene ontology in MQTL2.5. The intensity of the color increases from blue to red as the gene ontology (GO) term is enriched. The most enriched process in MQTL2.5 is the oxidation-reduction process, followed by electron transport chain and ethanol oxidation.

The 0.21cM interval of MQTL2.5 constitutes a 0.48kbp region in chromosome 2, where around 108 genes were annotated. Based on the gene ontology mapping and annotation for these genes (depicted as scatterplot in Figure 5), we found that the oxidation-reduction process, electron transport chain, ethanol oxidation and interstrand crosslink repair were among the most enriched biological processes in MQTL2.5. Around 12 genes were annotated with the oxidation-reduction process. Redox regulation was deemed to be crucial to alleviate the rapid oxidative burst that occurs as a consequence of stress imposition on plants during pathogen invasion [44]. Electron transport chain facilitates the generation of reactive oxygen species. While ethanol oxidation may not necessarily be linked to disease resistance, interstrand crosslink repair has been associated with DNA-related general housekeeping processes.

**Figure 6:**
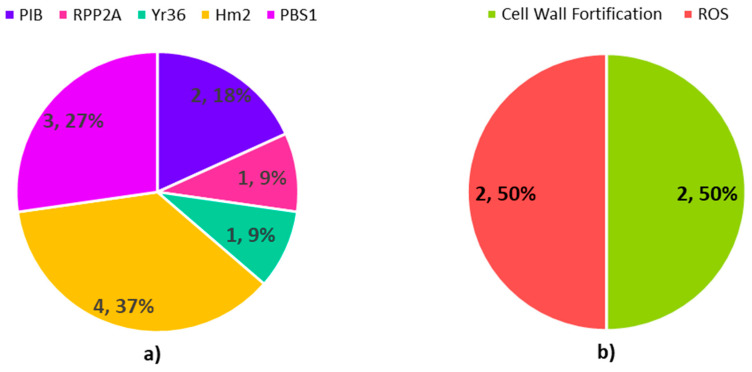
Pie chart representing (**a**) R-genes in MQTL2.5, (**b**) defense genes in MQTL2.5.

Further, around 11 resistance gene homologues were identified after a blast search against PRGdb (Figure 6a and Appendix A Table A1). This group of genes was described as disease resistance proteins, DUF640 domain containing proteins, OsWAK24, dehydrogenase, dihydroflavonol-4-reductase, cinnamoyl CoA reductase, serine/threonine-protein kinase, putative, ELMO/CED-12 family protein and pib. The full details on these genes are provided in the Appendix A Table A1. These genes were annotated as disease-resistant proteins and pib is analogous to the *Pib* gene, an NBS-LRR R-gene for *M. oryzae* in rice [45]. DUF640 domain containing protein is a match for the *RPP2A* resistance gene in *A. thaliana,* which demonstrates resistance against *Hyaloperonospora parasitica* [46]. OsWAK24 is a wall-associated receptor kinase with similarity to the *Yr36* R-gene in *Triticum turgidum* subsp. Dicoccoides [47]. Dehydrogenase, dihydroflavonol-4-reductase and cinnamoyl CoA reductase show similarity to the *Hm2* R-gene in *Zea mays* [48]. Serine/threonine-protein kinase and ELMO/CED-12 family protein are identical to PBS1 in *A. thaliana,* which is needed for RPS5 disease resistance protein-mediated plant defense [49] (Figure 6a and Appendix A Table A1).

In terms of defense genes, several genes are involved in cell wall fortification and reactive oxygen species (ROS) production and scavenging (Figure 6b and Appendix A Table A2). Around four copies of cinnamoyl-CoA reductase 1 have been implicated in lignin biosynthesis, which fortifies and strengthens the cell wall to provide the first line of defense against the penetration of the host by the pathogen. A study by Mutuku et al. (2019) demonstrated that, upon infection of *Striga hermonthica* in rice, the accumulation of metabolites related to the deposition of lignin was observed at the site of infection. Lignin makes up the cell wall of vascular plants and is induced upon biotic stress [50]. A rapid burst of ROS was observed as one of the earliest events after the imposition of stress [51]. Apart from this, four copies of protein LUTEIN DEFICIENT 5 were involved in oxidoreductase activity involving ROS. The production of ROS can trigger the defense signal and activate downstream genes. However, when above the threshold, ROS can be toxic to the host plant. Hence, to scavenge the excessive ROS, protein LUTEIN DEFICIENT 5 is required through the action of oxidoreductase activity [52] (Figure 6b and Appendix A Table A2).

#### 2.4.2. MQTL8.1

**Figure 7:**
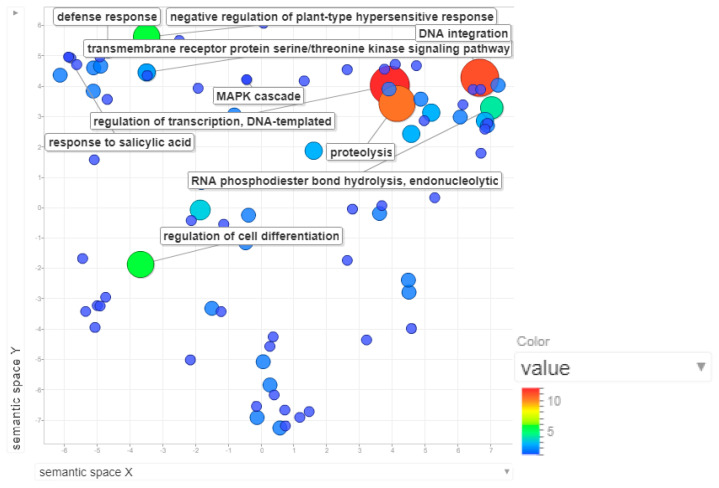
Scatterplot representing the enriched biological process gene ontology in MQTL8.1. The intensity of the color increases from blue to red as the GO term is enriched. The most enriched processes in MQTL8.1 are the regulation of transcription, DNA integration, proteolysis and negative regulation of plant type hypersensitive response.

MQTL8.1, with a confidence interval of 3.13cM, spans around 1.13kbp in chromosome 8. Functional analysis of this region yielded around 196 annotated genes. The most enriched biological processes in this MQTL are regulation of transcription, DNA integration, proteolysis and negative regulation of plant type hypersensitive response (Figure 7). Transcription factors govern various physiological and biochemical processes for an orderly series of events leading to defense in plants [53]. A high number of genes involved in DNA integration are transposable elements, which are largely repetitive elements in the plant genome that cause genome expansion [54]. Proteolysis by proteases not only helps in the detection of pathogen invasion but also induces defense response and activates defense signaling regulators [55]. The hypersensitivity response, which is manifested as cell death in the form of necrotic lesions, is important to contain further propagation of pathogens [56].

**Figure 8:**
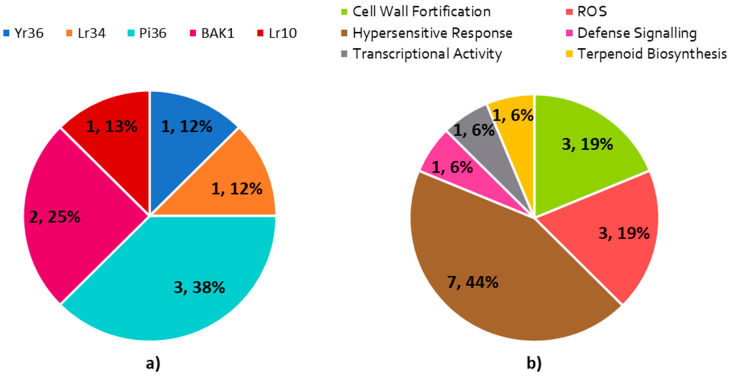
Pie chart representing (**a**) R-genes in MQTL8.1, (**b**) defense genes in MQTL8.1.

Around nine RGAs were found in the blast search against PRGdb, which are annotated as CGMC_MAPKCMGC_2_ERK.13, ABC-2 type transporter domain containing protein, RGH1A, mla1, BRASSINOSTEROID INSENSITIVE 1-associated receptor kinase 1 precursor and disease resistance protein RPM1. CGMC_MAPKCMGC_2_ERK.13 is analogous to the *Yr36* gene in *T. turgidum* [47]. ABC-2 type transporter domain containing protein shows similarity to *Lr34 in Triticum aestivum* [57]. RGH1A and mla1 is matched against *Pi36*, a rice R-gene for *M. oryzae* [58]. BRASSINOSTEROID INSENSITIVE 1-associated receptor kinase 1 precursor and disease resistance protein RPM1 are identical to *BAK1* in *A. thaliana* [59] (Figure 8a and Appendix A Table A3).

In addition, defense genes that are related to cell wall fortification, hypersensitive response, ROS, defense signaling, transcriptional activity and terpenoid biosynthesis were found in MQTL8.1. Two genes annotated as hydroxyproline-rich glycoprotein-like are plant wall glycoproteins that serve as barriers against pathogens. Cell wall hardening caused by these proteins was observed during pathogen infection, suggesting their involvement in the first line of defense [60]. Meanwhile, a gene annotated as O-methyltransferase, involved in the biosynthesis of lignin, was identified and noted for its involvement in cell wall fortification [61]. Aside from this, a gene annotated as mitogen-activated protein kinase 2 (MAPK2) is involved in MAPK cascades that serves as a central signaling pathway related to downstream defense processes [62] (Figure 8b and Appendix A Table A4).

Around seven copies of lesion stimulating disease protein-1 (LSD-1) involved in negative regulation of the plant hypersensitive response prevents the elicitation of the hypersensitive response. The silencing of this gene in rice has made the plant mimic disease lesion, which in turn upregulates the expression of PR-1 proteins [63]. A gene annotated as transposon protein, CACTA, En/Spm sub-class, has been implicated as being involved in transcriptional activity related to plant defense. Transposon is a repetitive element found throughout the genomes of organisms which are responsible for the expansion of the genome as they replicate the DNA sequence into other parts of the genome [54,64]. RIM2 elements in the transposon (CACTA family) were transcriptionally activated upon infection by *Magnaporthe grisea*, suggesting a hypothetical role in plant defense at the transcriptional level [65]. Around three genes annotated as reticuline oxidase are involved in oxidoreductase activity that serves to scavenge excess ROS and prevent cellular and molecular damage to plants [52]. Lastly, a gene annotated as uncharacterized protein LOC4344772 is involved in terpenoid biosynthesis. Terpenoids are compounds with antimicrobial activity that may be induced upon infection by the pathogen. An overexpressed rice terpene synthase gene (OsTPS19) led to increased resistance of rice against *M. oryzae* [66] (Figure 8b and Appendix A Table A4).

#### 2.4.3. MQTL9.1

**Figure 9:**
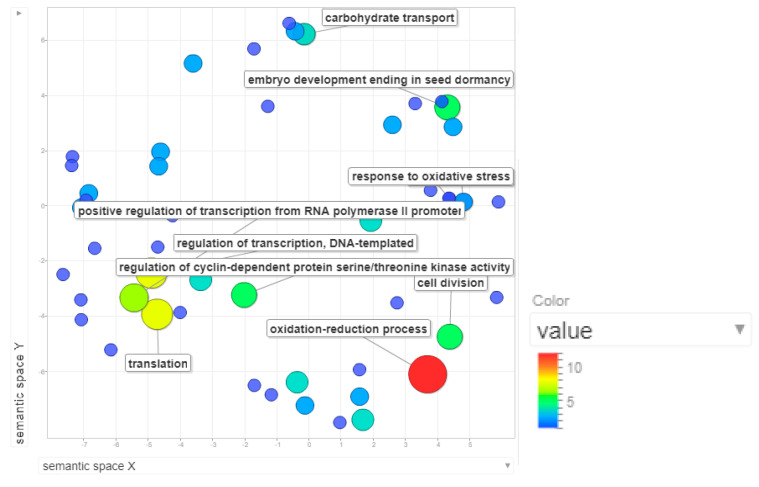
Scatterplot representing the enriched biological process gene ontology in MQTL9.1 The intensity of the color increases from blue to red as the GO term is enriched. The most enriched processes in MQTL9.1 are oxidation-reduction, translation, regulation of transcription, positive regulation of transcription from RNA polymerase II promoter and regulation of cyclin-dependent protein serine/threonine activity.

The 1.38cM interval of MQTL9.1 corresponds to the 0.46kb region in chromosome 9. The total number of genes annotated in MQTL9.1 via Blast2GO is 109. The top enriched biological processes are related to the oxidation-reduction process, translation, regulation of transcription, positive regulation of transcription from RNA polymerase II promoter and regulation of cyclin-dependent protein serine/threonine activity (Figure 9). Translation is a major biological process required for the translation of transcripts to protein. Positive regulation of transcription from RNA polymerase II is crucial to accelerate transcription. These two processes are perceived as general housekeeping processes. The involvement of cyclin dependent kinases in plant defense against pathogens is believed to be exhibited through its role in the transcriptional activation of a plant’s defense pathway [67].

**Figure 10:**
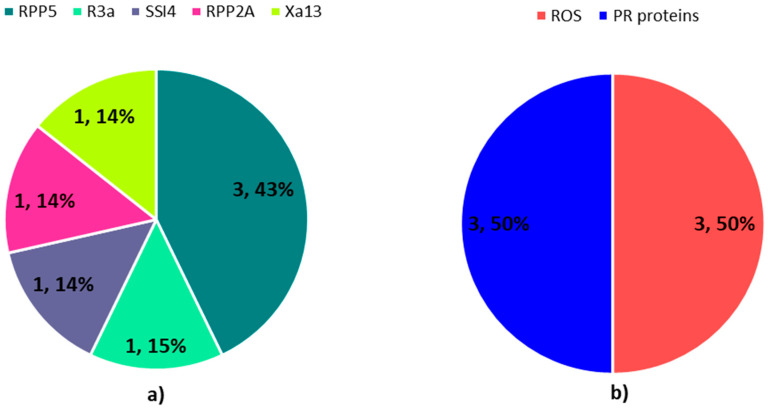
Pie chart representing (**a**) R-genes in MQTL9.1, (**b**) defense genes in MQTL9.1.

In addition, around seven RGAs, annotated as conserved hypothetical protein, expressed protein, OsFBX334-F-box domain containing protein and OsFBX335-F-box domain containing protein, were identified. The conserved hypothetical protein and OsFBX335-F-box domain containing protein are identical to the *RPP5* gene in *A. thaliana*. Three expressed proteins were matched to *R3a* (*Solanum tuberosum*), *RPP2A* (*A. thaliana*) and *Xa13* (*O. sativa*) genes, disease resistance genes against potato late blight, downy mildew and bacterial leaf blight, respectively [46,68,69]. OsFBX334-F-box domain containing protein is akin to SSI4, a TIR-NBS-LRR containing R-gene in *A. thaliana* [70] (Figure 10a and Appendix A Table A5).

Further, two pathogenesis-related (PR) proteins, namely glucan endo-1,3-beta-glucosidase, commonly known as β-1,3-glucanases (PR-2 protein), were identified in MQTL9.1 and are known to facilitate downstream defense processes. A copy of peroxidase, a PR-9 protein, was also identified. This protein plays a pivotal role in generating ROS that triggers the hypersensitivity response and restricts infection [71]. Three genes involved in oxidoreductase activity were chloroplast envelope quinone oxidoreductase homolog and L-ascorbate oxidase (Figure 10b and Appendix A Table A6).

## 3. Materials and Methods

### 3.1. Bibliographic Search and Data Mining

We conducted an exhaustive bibliographic search against all the published papers between 1995 to 2019 and compiled all the information on QTLs pertaining to resistance against RB, SHB and BLB in rice (*Oryza sativa* L.). The information includes parent population, type of mapping population (recombinant inbred line, RIL, and backcross, BC), size of population assayed, logarithm of odds (LOD), phenotypic variance (R2), molecular markers flanking the QTL along with its genetic position and the genetic position of the QTL interval. After careful examination of the compiled information, the studies that lacked the required information, such as the genetic position, LOD and phenotypic variance, were excluded. For those studies that provided p-values instead of LOD scores, a spreadsheet to convert p-values to LOD and vice versa by Nyholt et al. (2000) was used [72]. In total, around 27 studies were compiled for our current analysis and are summarized in Table 1.

### 3.2. Generation of Consensus Map

Following data compilation, two separate input files (map file and QTL file) were prepared for each study according to the user guide provided for BioMercator V4.2 [73] in txt. format. The files were then uploaded into BioMercator V4.2. To construct a consensus map, all the maps with the markers and QTLs were iteratively projected on a reference map produced by Temnykh et al. (2004) [74] in order of highest quality to lowest quality to ensure smooth arrangement of the markers. Any markers that did not comply (inverted) in terms of linkage were automatically discarded.

### 3.3. Meta-QTL Analysis

Following the construction of the consensus map and projection of the QTLs, meta-QTL analysis was performed on individual chromosomes to refine the QTL hotspot regions. To achieve this, the algorithm developed by Veyrieras was used, which involves a two-step analysis. In the first step, the best model (best number of meta-QTLs for a chromosome) is computed. The output for this step is produced in 3 files containing the computation data. The first file contains the computation data based on 5 criteria, namely Akaike Information Criterion (AIC), corrected Akaike Information Criterion (AICc), Akaike Information Criterion 3 (AIC3), Bayesian Information Criterion (BIC) and Approximate Weight of Evidence Criterion (AWE). These criteria evaluate the quality of the different models that are being compared, where the model with the lowest criterion value and 0 delta value is considered as the best model as it corresponds to the least information lost as compared to other models. For example, if model 4 has the lowest AIC value and 0 delta value, the suitable and precise number of meta-QTLs estimated for a particular chromosome will be 4. In the second file, the computation data from the previous file are summarized to display the best model suggested by each criterion. The best model has the majority of the suggested criteria. The third file comprises the summary of the clustering results. The second step involves setting up the appropriate parameters for the meta-analysis, including the number of meta-QTLs to be mapped. Here, we selected the suggested model from previous steps and kept the other parameters as default. Following the second step, another file was added into the task pane that contained the information of the meta-QTL analysis.

### 3.4. Functional Analysis

The selected meta-QTL regions were subjected to functional analysis to identify candidate genes related to the trait of interest. The physical positions of the markers flanking the meta-QTL regions were determined by Gramene [75] or by subjecting the primer sequence of the marker to nucleotide blast in NCBI [76] to identify the range of the sequence in the reference genome of Nipponbare [77]. Then, the nucleotide sequence within the region of interest was uploaded into Blast2GO [78] in fasta format for functional analysis. The description and the gene ontology (GO) of the list of genes within the region was then obtained. InterProScan [79] was used to determine the domains or repeats found in the genes. The gene ontology enrichments were visualized using ReviGO, where the intensity of the bubble color increases from blue to red as the GO term is enriched [80]. The fasta sequence of the QTL region was also subjected to blast against the plant disease resistance gene database (PRGDB) to determine the resistance gene analogs [81]. The positions and frequency of the meta-QTLs on the 12 rice chromosomes were visualized using ClicO FS [82].

## 4. Conclusions

A vast number of studies have been conducted to determine QTLs associated with rice blast, sheath blast and bacterial leaf blight across different regions and varieties. However, meta-QTL analyses for these QTLs have not been reported to date. Considering this, we have integrated all the QTL mapping studies pertaining to RB, SHB and BLB resistance to investigate the consensus between these diseases and performed a meta-analysis to refine the QTL clusters. A total of 48 meta-QTLs were obtained through the meta-analysis using BioMercator V4.2, which was further reduced to 36 meta-QTLs after filtering out the undersaturated regions. In terms of resistance against multiple diseases, only three meta-QTLs were eligible for multiple resistance, MQTL2.5, MQTL8.1 and MQTL9.1, which had 19, 12 and 10 QTLs, respectively, as members. A considerable number of R-genes and defense genes were identified within these meta-QTL regions through functional analysis. The R-genes were found to confer resistance against a wide array of pathogens, including fungi and bacteria, that go beyond the microorganisms’ links to the diseases elucidated in this study. The defense genes identified were associated with diverse functions such as cell wall fortification, defense signaling, reactive oxygen species, anti-microbial activity, biosynthesis of secondary metabolites and transcriptional activity. The R-genes recognize the pathogen and relay the signals to activate defense genes, which elicit broad spectrum resistance against the pathogen. The information on the markers associated with these meta-QTL regions and genes occupying the region will be useful in breeding for resistance against multiple diseases.

## 5. Copyright

The consensus genetic map is protected as genetic mapping of QTLs associated with rice diseases (F.2939).

## Figures and Tables

**Table 1:** Summary of QTL mapping studies employed for the generation of consensus map (RB: rice blast, SHB: sheath blight, BLB: bacterial leaf blight, F: filial generation, RILs: recombinant inbred lines, DH: double haploid, BC: backcross, NIL: near isogenic line).

Disease	Parents	Cross Type	No of Markers Used	Type of Marker	Population Size	No. of QTLs	References
RB	Mahsuri x Pongsu Seribu	F3	63	SSR	300	40	[4]
SHB	Tetep x HP2216	RILs, F2:10	126	SSR	127	12	[5]
RB	ZhenShan97 x Minghui63	RILs	127	SSR, RFLP	241	12	[6]
BLB	Azucena x IR64	RILs	226	SSR	172	14	[38]
SHB	RSB03 x HH1B	RILS, F2:7 and F2:8	123	SSR, InDel	121	28	[7]
RB	Owarihatamochi x Nipponbare	BC1F3	118	SSR, RFLP	82	4	[8]
SHB	Teqing x Lemont	RILs		SSR, RFLP	240	2	[9]
SHB	Jasmine85 x Lemont	RILs	199	SSR	227	9	[10]
SHB	Teqing x Lemont	Bulk F4	113	RFLP	255	6	[11]
BLB	Teqing x Lemont	RILs, F13	279	SSR, RFLP	292	23	[12]
BLB	Teqing x Lemont	DH	176	RFLP	125	28	[12]
RB	ZhenShan x MingHui	RILs, F10	227	SSR, RFLP	241	22	[13]
SHB	Jasmine85 x Lemont	RILs, F5	199	SSR	250	12	[14]
SHB	Jasmine95 x Lemont	RILs, F5	199	SSR	250	14	[15]
SHB	Jasmine85 x Lemont	F2 clonal	94	SSR, RFLP	128	3	[16]
RB	KHZ x TAM	F2 clonal	74	SSR	192	7	[17]
SHB	Rosemont x Pecos	F2:3	149	SSR	279	8	[18]
RB	KDML105 x CT9993-5-10-M	RILs, F8	121	SSR, RFLP	141	10	[19]
RB	Teqing x Lemont	RILs, F8	173	RFLP	280	9	[20]
RB	Moroberekan x C039	RILs, F7	171	RFLP	300	10	[21]
BLB	TN1 x SA0423	F2	151	SSR, InDel	184	3	[22]
SHB	Yangdao4 x Lemont	F2 and F2:3	180	SSR, InDel	568	21	[23]
SHB	Baiyeqiu x Maybelle	DH	282	SSR	251	4	[24]
SHB	ARC10531 x BPT-5204	BC1F2	70	SSR	150	9	[25]
SHB	Teqing x Lemont	NIL, BC6F1	114	SSR, InDel	1200	1	[26]
SHB	CJ06 x TN1	DH	214	SSR	116	16	[27]
SHB	Jasmine85 x Lemont	F2 clonal	118	SSR, RFLP	128	8	[28]

**Table 2:** Meta-QTLs associated with resistance against RB, SHB and BLB along with other information such as the range of LOD and phenotypic variance of initial QTL and the confidence interval of the meta-QTL.

Chromosome	MQTL	Number of QTL(s) Involved	Confidence Interval (cM)	Diseases Involved	LOD	R2 (%)
1	MQTL1.1	11	0.49	SHB, RB	1–12.3	1.6–42.6
	MQTL1.2	8	2.37	SHB, RB	2.2–8.18	2–4.83
	MQTL1.3	2	2.67	BLB, RB	2.17–5.7	0.89–6.84
	MQTL1.4	3	0.4	BLB, RB	5.07–30.6	13.4–36.4
2	MQTL2.1	11	1.18	SHB, BLB	2.23–6.3	1.75–22.1
	MQTL2.2	3	4.57	SHB, RB	3.1–4.14	0.93–8.2
	MQTL2.3	4	3.97	SHB	2.6–4.1	3.2–6.64
	MQTL2.4	1	0.22	BLB	1.35–27.8	3–44.2
	MQTL2.5	19	0.21	SHB, RB	2.85	
	MQTL2.6	1	1.18	RB	5.88	2.13
3	MQTL3.1	7	2.41	SHB, RB	2.97–17.61	0.229–9.96
	MQTL3.2	8	0.12	SHB, RB	1.9–4.04	3.2–15.9
	MQTL3.3	13	0.5	SHB, BLB	1.54–7.57	0.63–31.53
	MQTL3.4	5	2.34	RB	2.34–5.36	10.9–17.61
	MQTL3.5	2	0.07	SHB	1.65–2.8	3–5.57
4	MQTL4.1	1	1.56	RB	6.24	14.45
	MQTL4.2	2	5.09	RB, SHB	2.7–3.35	4.15–5.6
	MQTL4.3	1	5.51	RB	5.86	1.99
	MQTL4.4	1	11.87	BLB	2.26	0.91–0.91
	MQTL4.5	3	7.79	SHB, BLB	3.403–3.8	10–15.7
	MQTL4.6	2	1.73	SHB, RB	3–11	17.53–29.4
5	MQTL5.1	3	5.01	SHB, RB	4.4–6.1	0.24–5.1
	MQTL5.2	5	2.8	BLB	3.4–6.7	1.01–11.5
	MQTL5.3	2	7.73	SHB	3.66	10
	MQTL5.4	6	0.3	SHB	2.03–6.8	5.7–15.7
6	MQTL6.1	12	0.76	RB	0.87–13.7	0.28–40
	MQTL6.2	4	5.63	RB, BLB	2.66–13.2	4.6–9.58
7	MQTL7.1	7	0.13	RB, SHB, BLB	2.6–16.01	2.3–36.6
	MQTL7.2	1	0.1	RB	2.7	
	MQTL7.3	7	0.98	RB	2–8.8	2.1–21.76
	MQTL7.4	2	23.94	RB	3	11–12
8	MQTL8.1	12	3.13	RB, SHB, BLB	3–15.04	0.165–16.71
	MQTL8.2	1	4.27	RB	4.5–4.5	7.22–7.22
	MQTL8.3	2	1.82	RB, BLB	3.51–6.11	1.43–10.49
	MQTL8.4	5	2.99	SHB	3.5–6	5.8–23.5
9	MQTL9.1	10	1.38	SHB, BLB, RB	2–5.9	3–12.9
	MQTL9.2	4	0.04	SHB, RB	4.44–10.1	0.99–4.75
	MQTL9.3	1	0.07	SHB	6.9–6.9	2.5
	MQTL9.4	9	0	SHB, RB	2.27–19.9	2.82–27.2
10	MQTL10.1	6	19.78	RB	1.04–7.7	3–5
	MQTL10.2	2	0.07	BLB	2.68–5.04	0.77–1.13
11	MQTL11.1	7	10.78	SHB	3.7–6.19	1.56–15.19
	MQTL11.2	2	8.44	SHB	3–4.38	9.77–21.59
	MQTL11.3	8	4.82	BLB, RB	1.39–36.78	3–67.9
	MQTL11.4	7	1.76	BLB, RB	2.66–26.6	3.53–26.53
12	MQTL12.1	3	0.63	SHB	3.31–4.2	6.99–11.95
	MQTL12.2	1	10.74	SHB	3.12	10.49
	MQTL12.3	3	3.97	RB	4.52–5.49	7.2–8.7
	MQTL12.4	6	4.17	RB	4.33–20.1	4.16–13.7
	MQTL12.5	1	10.66		3	9.15

## Abbreviations

MDPI	Multidisciplinary Digital Publishing Institute
QTL	quantitative trait loci
MQTL	Meta-QTL
R-gene	resistance gene
RB	rice blast
SHB	sheath blight
BLB	bacterial leaf blight

## Appendix A

**Table A1:** List of R-genes in MQTL2.5.

Locus ID	Gene Description	Locus ID PRGdb
LOC_Os02g57305.1	disease resistance protein, putative, expressed	150957_PIB
LOC_Os02g56610.1	DUF640 domain containing protein, putative, expressed	170018_RPP2A
LOC_Os02g56630.1	OsWAK24—OsWAK receptor-like protein kinase, expressed	170039_Yr36
LOC_Os02g56680.1	dehydrogenase, putative, expressed	1499_Hm2
LOC_Os02g56690.1	dihydroflavonol-4-reductase, putative, expressed	1499_Hm2
LOC_Os02g56700.1	dehydrogenase, putative, expressed	1499_Hm2
LOC_Os02g56720.2	cinnamoyl CoA reductase, putative, expressed	1499_Hm2
LOC_Os02g57080.1	serine/threonine-protein kinase, putative, expressed	170031_PBS1
LOC_Os02g57080.2	serine/threonine-protein kinase, putative, expressed	170031_PBS1
LOC_Os02g57160.1	ELMO/CED-12 family protein, putative, expressed	170031_PBS1
LOC_Os02g57310.1	pib, putative, expressed	150957_PIB

**Table A2:** List of defense genes in MQTL2.5.

Locus ID	Gene Description	Gene Ontology
LOC_Os02g56680.1	cinnamoyl-CoA reductase 1	F:protein binding; C:cytoplasm; P:defense response; P:lignin biosynthetic process; F:oxidoreductase activity, acting on the CH-OH group of donors, NAD or NADP as acceptor; F:cinnamoyl-CoA reductase activity; F:coenzyme binding; P:oxidation-reduction process
LOC_Os02g56690.1	cinnamoyl-CoA reductase 1-like	F:protein binding; C:cytoplasm; P:defense response; P:lignin biosynthetic process; F:oxidoreductase activity, acting on the CH-OH group of donors, NAD or NADP as acceptor; F:cinnamoyl-CoA reductase activity; F:coenzyme binding; P:oxidation-reduction process
LOC_Os02g56700.1	cinnamoyl CoA reductase	F:protein binding; C:cytoplasm; P:defense response; P:lignin biosynthetic process; F:oxidoreductase activity, acting on the CH-OH group of donors, NAD or NADP as acceptor; F:cinnamoyl-CoA reductase activity; F:coenzyme binding; P:oxidation-reduction process
LOC_Os02g56720.2	cinnamoyl-CoA reductase 1-like	F:protein binding; C:cytoplasm; P:defense response; P:lignin biosynthetic process; F:oxidoreductase activity, acting on the CH-OH group of donors, NAD or NADP as acceptor; F:cinnamoyl-CoA reductase activity; F:coenzyme binding; P:oxidation-reduction process
LOC_Os02g57290.1	protein LUTEIN DEFICIENT 5, chloroplastic	F:iron ion binding; C:chloroplast envelope; F:carotene beta-ring hydroxylase activity; P:xanthophyll biosynthetic process; F:oxidoreductase activity, acting on paired donors, with incorporation or reduction of molecular oxygen; F:heme binding; P:oxidation-reduction process
LOC_Os02g57290.4	protein LUTEIN DEFICIENT 5, chloroplastic	F:iron ion binding; C:chloroplast envelope; F:carotene beta-ring hydroxylase activity; P:xanthophyll biosynthetic process; F:oxidoreductase activity, acting on paired donors, with incorporation or reduction of molecular oxygen; F:heme binding; P:oxidation-reduction process
LOC_Os02g57290.2	protein LUTEIN DEFICIENT 5, chloroplastic	F:iron ion binding; C:chloroplast envelope; F:carotene beta-ring hydroxylase activity; P:xanthophyll biosynthetic process; F:oxidoreductase activity, acting on paired donors, with incorporation or reduction of molecular oxygen; F:heme binding; P:oxidation-reduction process
LOC_Os02g57290.3	protein LUTEIN DEFICIENT 5, chloroplastic	F:iron ion binding; C:chloroplast envelope; F:carotene beta-ring hydroxylase activity; P:xanthophyll biosynthetic process; F:oxidoreductase activity, acting on paired donors, with incorporation or reduction of molecular oxygen; F:heme binding; P:oxidation-reduction process

**Table A3:** List of R-genes in MQTL8.1.

Locus ID	Gene Description	Locus ID PRGdb
LOC_Os08g06060.1	CGMC_MAPKCMGC_2_ERK.13—CGMC includes CDA, MAPK, GSK3 and CLKC kinases, expressed	170039_Yr36
LOC_Os08g07010.1	ABC-2 type transporter domain containing protein, expressed	161470_Lr34
LOC_Os08g07330.2	RGH1A, putative, expressed	161436_Pi36
LOC_Os08g07340.1	mla1, putative, expressed	161436_Pi36
LOC_Os08g07370.1	RGH1A, putative, expressed	161436_Pi36
LOC_Os08g07390.1	BRASSINOSTEROID INSENSITIVE 1-associated receptor kinase 1 precursor, putative, expressed	170016_BAK1
LOC_Os08g07774.1	disease resistance protein RPM1, putative, expressed	161439_Lr10
LOC_Os08g07774.2	disease resistance protein RPM1, putative, expressed	170016_BAK1

**Table A4:** List of defense genes in MQTL8.1.

Locus ID	Gene Description	Gene Ontology
LOC_Os08g06060.1	mitogen-activated protein kinase 2	P:MAPK cascade; F:MAP kinase activity; F:protein tyrosine kinase activity; F:ATP binding; C:nucleus; C:cytoplasm; P:regulation of gene expression; P:peptidyl-tyrosine phosphorylation
LOC_Os08g06100.1	O-methyltransferase	C:nucleus; C:cytoplasm; C:plasma membrane; C:plasmodesma; P:response to wounding; P:response to ethylene; P:response to salicylic acid; P:lignin biosynthetic process; F:acetylserotonin O-methyltransferase activity; P:melatonin biosynthetic process; F:luteolin O-methyltransferase activity; F:quercetin 3-O-methyltransferase activity; P:methylation; F:myricetin 3′-O-methyltransferase activity; P:response to hydrogen peroxide; F:protein dimerization activity; F:caffeate O-methyltransferase activity; P:flavonol biosynthetic process; F:quercetin 3′-O-methyltransferase activity
LOC_Os08g06170.1	reticuline oxidase	F:oxidoreductase activity; P:oxidation-reduction process; F:FAD binding
LOC_Os08g06180.1	reticuline oxidase	F:oxidoreductase activity; P:oxidation-reduction process; F:FAD binding
LOC_Os08g06190.1	reticuline oxidase	F:oxidoreductase activity; P:oxidation-reduction process; F:FAD binding
LOC_Os08g06280.1	protein LSD1	C:nucleus; P:negative regulation of plant-type hypersensitive response; P:regulation of cell differentiation
LOC_Os08g06280.2	protein LSD1	C:nucleus; P:negative regulation of plant-type hypersensitive response; P:regulation of cell differentiation
LOC_Os08g06280.3	protein LOL1-like	C:nucleus; P:negative regulation of plant-type hypersensitive response; P:regulation of cell differentiation
LOC_Os08g06280.4	protein LSD1	C:nucleus; P:negative regulation of plant-type hypersensitive response; P:regulation of cell differentiation
LOC_Os08g06280.7	protein LSD1	C:nucleus; P:negative regulation of plant-type hypersensitive response; P:regulation of cell differentiation
LOC_Os08g06280.8	protein LSD1	C:nucleus; P:negative regulation of plant-type hypersensitive response; P:regulation of cell differentiation
LOC_Os08g06280.9	Protein LSD1	C:nucleus; P:negative regulation of plant-type hypersensitive response; P:regulation of cell differentiation
LOC_Os08g06380.1	cellulose synthase-like CslF6	C:Golgi membrane; C:trans-Golgi network; C:plasma membrane; P:plant-type primary cell wall biogenesis; C:integral component of membrane; F:cellulose synthase (UDP-forming) activity; P:cellulose biosynthetic process; F:mannan synthase activity; P:cell wall organization; P:mannosylation
LOC_Os08g06970.1	hydroxyproline-rich glycoprotein-like	F:serine-type endopeptidase activity; P:proteolysis
LOC_Os08g07350.1	transposon protein, putative, CACTA, En/Spm sub-class	P:proteolysis; P:defense response; F:cysteine-type peptidase activity
LOC_Os08g07430.1	uncharacterized protein LOC4344772	F:magnesium ion binding; C:cytoplasm; P:defense response; F:terpene synthase activity; C:integral component of membrane; P:terpenoid biosynthetic process; F:beta-sesquiphellandrene synthase activity

**Table A5:** List of R-genes in MQTL9.1.

Locus ID	Gene Description	Locus ID PRGdb
LOC_Os09g32400.1	conserved hypothetical protein	1493_RPP5
LOC_Os09g32580.1	expressed protein	147_R3a
LOC_Os09g32600.1	OsFBX334—F-box domain containing protein, expressed	161441_SSI4
LOC_Os09g32610.1	expressed protein	170018_RPP2A
LOC_Os09g32860.1	OsFBX335—F-box domain containing protein, expressed	1493_RPP5
LOC_Os09g32860.2	OsFBX335—F-box domain containing protein, expressed	1493_RPP5
LOC_Os09g32992.1	expressed protein	161451_Xa13

**Table A6:** List of defense genes in MQTL9.1.

Locus ID	Gene Description	Gene Ontology
LOC_Os09g32390.1	transposon protein, putative, mutator sub-class	F:aspartic-type endopeptidase activity; F:hydrolase activity, hydrolyzing O-glycosyl compounds; P:carbohydrate metabolic process; P:proteolysis; F:zinc ion binding; F:oxidoreductase activity, acting on the CH-OH group of donors, NAD or NADP as acceptor; F:NAD binding; P:oxidation-reduction process
LOC_Os09g32570.1	chloroplast envelope quinone oxidoreductase homolog	F:oxidoreductase activity; P:oxidation-reduction process
LOC_Os09g32620.1	chloroplast envelope quinone oxidoreductase homolog	F:oxidoreductase activity; P:oxidation-reduction process
LOC_Os09g32952.1	L-ascorbate oxidase	F:copper ion binding; C:extracellular region; F:oxidoreductase activity; P:oxidation-reduction process
LOC_Os09g32964.1	peroxidase 73	F:peroxidase activity; C:extracellular region; P:response to oxidative stress; C:integral component of membrane; F:heme binding; P:hydrogen peroxide catabolic process; F:metal ion binding; P:oxidation-reduction process; P:cellular oxidant detoxification
LOC_Os09g32550.1	probable glucan endo-1,3-beta-glucosidase A6	F:hydrolase activity, hydrolyzing O-glycosyl compounds; P:carbohydrate metabolic process; C:anchored component of plasma membrane
LOC_Os09g32550.2	probable glucan endo-1,3-beta-glucosidase A6	F:hydrolase activity, hydrolyzing O-glycosyl compounds; P:carbohydrate metabolic process; C:anchored component of plasma membrane
